# Biomimetic Mineralization on a Macroporous Cellulose-Based Matrix for Bone Regeneration

**DOI:** 10.1155/2013/452750

**Published:** 2013-09-19

**Authors:** Odeta Petrauskaite, Pedro de Sousa Gomes, Maria Helena Fernandes, Gintaras Juodzbalys, Arturas Stumbras, Julius Maminskas, Jolanta Liesiene, Marco Cicciù

**Affiliations:** ^1^Department of Organic Technology, Kaunas University of Technology, Radvilenu pl. 19, 50254 Kaunas, Lithuania; ^2^Laboratory for Bone Metabolism and Regeneration, Faculty of Dental Medicine, University of Porto, Rua Dr. Manuel Pereira da Silva, 4200-392 Porto, Portugal; ^3^Department of Oral and Maxillofacial Surgery, Lithuanian University of Health Sciences, Eiveniu str. 2, 50009 Kaunas, Lithuania; ^4^Human Pathology Department, Dental School, University of Messina, Messina IT, Policlinico G. Martino, Via Consolare Valeria, 98100 Messina, Italy

## Abstract

The aim of this study is to investigate the biomimetic mineralization on a cellulose-based porous matrix with an improved biological profile. The cellulose matrix was precalcified using three methods: (i) cellulose samples were treated with a solution of calcium chloride and diammonium hydrogen phosphate; (ii) the carboxymethylated cellulose matrix was stored in a saturated calcium hydroxide solution; (iii) the cellulose matrix was mixed with a calcium silicate solution in order to introduce silanol groups and to combine them with calcium ions. All the methods resulted in a mineralization of the cellulose surfaces after immersion in a simulated body fluid solution. Over a period of 14 days, the matrix was completely covered with hydroxyapatite crystals. Hydroxyapatite formation depended on functional groups on the matrix surface as well as on the precalcification method. The largest hydroxyapatite crystals were obtained on the carboxymethylated cellulose matrix treated with calcium hydroxide solution. The porous cellulose matrix was not cytotoxic, allowing the adhesion and proliferation of human osteoblastic cells. Comparatively, improved cell adhesion and growth rate were achieved on the mineralized cellulose matrices.

## 1. Introduction

Several bone grafts techniques are currently available for favouring regenerative processes on bone trauma, or for promoting healing between two bones across a diseased joint, and also for having new bone formation on site affected by disease, infection, or resection. Nowadays, great attention is focused on polymer/ceramic three-dimensional scaffolds for bone tissue regeneration [1–7]. It is well known that by choosing an appropriate polymer [8] and ceramic, for example, hydroxyapatite (HA) due to its excellent osteoconductivity, biocompatibility, and bioactivity [9], it is possible to fabricate well functionalizing scaffolds. This composite material must be nontoxic, compatible with the surrounding biological systems, and biodegradable. The scaffold has to be a 3D interconnected porous structure capable of promoting cell adhesion, proliferation and vascularization, and enabling a controlled supply of bioactive substances, which may influence the behaviour of incorporated or ingrown cells [10, 11].

The cellulose matrix shares a number of these advantages. It is an abundant, renewable, biocompatible, non-toxic, and biodegradable polymer [12]. Moreover, it has good mechanical properties because of the strong hydrogen bonding between the cellulose chains [13]. However, it has no bioactivity within the bone tissue [14]. Several studies have carried out research to establish a direct bond between developed material and natural bone tissue: this consists of the development of a hydroxyapatite layer by means of biomimetic mineralization [9, 11, 15]. As such, a simulated electrolyte body fluid solution (SBF) with ion concentrations similar to those of human blood plasma is used. An increased concentration of calcium ions is also required as these accelerate the nucleation rate of the hydroxyapatite crystals.

To date, covering scaffold surfaces with hydroxyapatite layer by the biomimetic route for the bone-bonding ability continues to be of great interest. Polymers with hydrophilic polar (e.g., hydroxyl, carboxyl, and silanol) groups are used due to their capacity to induce apatite nucleation [16–19]. Hong et al. [19] have revealed that bacterial cellulose (BC) and hydroxyapatite composites could be prepared by soaking BC in CaCl_2_ solution prior to the biomimetic mineralization. However, introduced carboxyl functional groups on BC by TEMPO ((2,2,6,6-tetramethylpiperidine-1-oxyl)-mediated oxidation) and combined with calcium ions could enhance the rate of apatite nucleation as described by Nge and Sugiyama [20]. Greater numbers of smaller sized crystals grew on BC-TEMPO-Ca than did on native BC. Leonor et al. [21] successfully prepared bioactive chitosan microparticles with bone-bonding properties by introducing silanol groups combined with calcium ions, which were then soaked in a simulated body fluid. An apatite layer formed on their surfaces just under one day.

Consistent bone regeneration for the treatment of combined vertical and horizontal defects without the application of large bone grafts, exogenous growth factors, or cells still remains a challenge for clinicians and surgeons.

Several bone substitutes are currently available like carriers or scaffold. However, the effectiveness osteoconductivity property of the bone graft is still related with the microstructure of each material, and autogenous bone still remains the “gold standard.” The study about bone substitutes as a valid alternative to autogenous bone grafts is connected to several clinical challenges. Clinicians and surgeons would eliminate the need to harvest bone from body sites when performing oral and maxillofacial regenerative surgery and the patient's pain and discomfort associated to these procedures [3, 4, 6, 16].

In this study, a macroporous cellulose matrix with an improved biological profile and performed biomimetic mineralization to induce hydroxyapatite formation was prepared. Precalcification of the matrix was done by means of three different methods: (i) treating the samples with CaCl_2_ and (NH_4_)_2_HPO_4_ solutions; (ii) storing the carboxymethylated cellulose matrix in a saturated calcium hydroxide solution; (iii) treating the matrix with a calcium silicate solution. In addition, the biological performance of the porous cellulose matrix and the developed mineralized cellulose subtracts was evaluated with human osteoblastic cells regarding cell adhesion, morphology, and proliferation. Final aim of the paper is to investigate how this cellulose matrix could be predictable used for future application on large bone defects. The obtaining of a valid carrier or scaffold may be helpful for clinicians avoiding autogenous bone graft for other body sites reducing patient's discomfort, cost, and morbidity.

## 2. Materials and Methods

### 2.1. Materials

For this study, cellulose diacetate (DAC, 55% bond acetic acid) was obtained from Roshal (Russia). MG63 cells were obtained from the American Type Culture Collection (ATCC CRL-1427). Ammonia water (NH_3_·H_2_O, 25%) and acetone (CH_3_COCH_3_) were purchased from Stanlab (Poland). Ethanol (C_2_H_5_OH, 96.3%) was purchased from Stumbras (Lithuania). All other reagents were purchased from the Sigma-Aldrich Company.

### 2.2. Preparation of a Porous Cellulose Matrix

Cellulose-based gel was prepared by the regeneration of cellulose from cellulose diacetate (DAC) according to the patent [22]. For this purpose, 25 g of DAC was dissolved in 265 mL of an acetone-ammonia water mixture and conserved until a solid gel was formed. The gel was thoroughly rinsed with water. In order to create the highly porous matrix, cylindrical gel samples, with a diameter of about 12 mm and a thickness of 6 mm, were washed with the ethanol-water solution (1 : 4 v/v) and lyophilized in the *Christ ALPHA 2-4 LSC* freeze dryer.

### 2.3. Porosity

The porosity of the cellulose matrix was determined by the liquid displacement method [23]. Ethanol was used as a displacement liquid because it penetrates easily into the pores and does not cause shrinking or swelling of the matrix. The dehydrated sample (approx. 0.1 g) was placed in a graduated cylinder containing 4 mL (*V*_1_) of ethanol and stored at 37°C for 6 hrs. Before weighting, the tightly sealed cylinder containing the immersed sample was placed into an ultrasonic bath for 10 minutes to force ethanol into the pores of the polymer matrix. The total volume was then recorded as *V*_2_. Subsequently, the sample was taken out, and the volume of ethanol was recorded as *V*_3_. The porosity (*P*) of the cellulose matrix was calculated using the following:

(1) $$\begin{array}{c} P\left(\%\right)=\frac{V_{1}-V_{3}}{V_{2}-V_{3}}\times 100. \end{array}$$

### 2.4. Water Retention

Water retention of the prepared matrix was determined using phosphate buffer solution (pH 7.4) in order to simulate physiological conditions. The dehydrated sample (approx. 0.1 g (*W*_1_)) was immersed in a phosphate buffer solution at 37°C for 24 hrs. Before weighting, the bottle containing the sample was placed into an ultrasonic bath for 10 minutes to ensure that every pore was filled with the solution. Lastly, the sample was wiped with filter paper and weighted as *W*_2_. Water retention (WR) was then defined by:

(2) $$\begin{array}{c} \text{WR}\left(\%\right)=\frac{W_{2}-W_{1}}{W_{1}}\times 100. \end{array}$$

### 2.5. Incubation of Cellulose in CaCl_2_ and (NH_4_)_2_HPO_4_ Solutions

The precalcification and formation of an amorphous calcium phosphate layer, for further conversion to hydroxyapatite in a simulated body fluid, were carried out by sequentially immersing the dehydrated cellulose samples (approx. 0.5 g) in 50 mL of 0.2 mol L^−1^ CaCl_2_ solution and 50 mL of 0.2 mol L^−1^ (NH_4_)_2_HPO_4_ solution at room temperature for 1.5 hrs per step. After soaking, the samples were rinsed with distilled water and dried at 105°C for 24 hrs.

### 2.6. Carboxymethylation of the Cellulose Matrix and Treatment with a Saturated Calcium Hydroxide Solution

The cellulose matrix was carboxymethylated with chloroacetic acid in the presence of sodium hydroxide. For this purpose, 1 g of dehydrated cellulose was immersed in a mixture of 2.75 mL of 40% NaOH and 30 mL of ethanol. The mixture was heated to 50°C, and 1.29 g of chloroacetic acid was added. Carboxymethylation was performed at 60°C for 2.5 hrs. The reaction product was washed with distilled water until neutral pH was obtained and then lyophilized. The amount of carboxymethyl groups was determined using the Back Titration Method as detailed in the literature [24].

Furthermore, the activation of the carboxymethylated matrix was performed by immersing samples (approx. 0.5 g) into 50 mL of a saturated Ca(OH)_2_ solution at 0°C for 72 hrs [25]. After incubation, samples were rinsed with distilled water and dried at 105°C for 24 hrs.

### 2.7. Cellulose Treatment with Calcium Silicate Solution

The silanol groups were incorporated into the cellulose matrix by soaking approximately 0.5 g of the cellulose matrix in 50 mL of the calcium silicate solution [26] with ratio Si(OC_2_H_5_)_4_ : H_2_O : C_2_H_5_OH : HCl : CaCl_2_ 1 : 4 : 4 : 0.014 : 0.2 w/w for 3 hrs at 37°C. Polymer modification was followed by tetraethoxysilane hydrolysis and condensation reactions. After separation from the solution, the samples were dried at 105°C to a constant weight.

### 2.8. Biomimetic Mineralization

In vitro biomimetic mineralization was carried out by immersing the samples into a simulated body fluid (SBF) with a concentration factor of 1.5 (1.5x SBF) and the following ion concentrations [10^−3 ^mol L^−1^]: Na^+^ = 213.0; K^+^ = 7.5; Ca^2+^ = 3.75; Mg^2+^ = 2.25; Cl^−^ = 221.7; HPO_4_^2−^ = 1.5; HCO_3_^−^ = 6.3; SO_4_^2−^ = 0.75. The 1.5x SBF solution was prepared by dissolving the calculated amounts of NaCl, KCl, CaCl_2_, MgCl_2_·6H_2_O, NaHCO_3_, K_2_HPO_4_·3H_2_O, Na_2_SO_4_ in distilled water. The solution was buffered with Tris/HCl to pH 7.4 at 37°C. The samples were incubated in 1.5x SBF at 37°C for up to 14 days. The electrolyte solution was refreshed once a week. After separation from the solution, the samples were rinsed with distilled water and dried. The mass increase (MI) was calculated using:

(3) $$\begin{array}{c} \text{MI}\left(\%\right)=\frac{m_{1}-m_{0}}{m_{0}}\times 100, \end{array}$$

where *m*_0_ and *m*_1_ are the masses of the samples before and after the mineralization process, respectively.

### 2.9. Characterization

Infrared spectroscopy (IR) was used to analyse the chemical structure of cellulose before and after modification. Four mg of cellulose was mixed with 200 mg of KBr for the preparation of transparent pellets. All spectra were recorded in the range from 4000 to 400 cm^−1^ on a Perkin Elmer *FT-IR* spectrometer.

X-ray diffraction (XRD) was used for identification of the calcium phosphate phase. Diffraction patterns were recorded on a DRON-6 using a Cu K_*α*_ radiation at 30 kV and 20 mA.

A high-resolution field emission scanning electron microscope FEI Quanta 200 FEG with a Schottky type electron gun was used to observe the morphology of polymer and to detect hydroxyapatite.

### 2.10. In Vitro Biological Evaluation

In vitro biological evaluation was performed using MG63 osteoblastic cells. Cells were cultured until the adequate confluence (around 80%), in the *α*-MEM culture medium containing 10% fetal bovine serum, 100 *μ*g/mL penicillin, 10 IU/mL streptomycin, 2.5 *μ*g/mL fungizone, and 50 *μ*g/mL ascorbic acid, at 37°C, in a humidified atmosphere of 5% CO_2_ in air. For a subculture, the cell monolayer was washed twice with a phosphate-buffered saline (PBS) and incubated with a trypsin-EDTA solution (0.05% trypsin, 0.25% EDTA) for 5 minutes at 37°C to ensure cell detachment. Cells were followed counting by a hemocytometer and resuspended in the culture medium. The cell suspension was used in the biological evaluation of the cellulose porous scaffolds.

The scaffolds—the cellulose matrix (control) and biomimetic HA-covered cellulose—were placed on the bottom of the wells of 24-well culture plates (one scaffold/well) and were seeded with MG63 osteoblastic cells (10^5^ cells cm^2^). Seeded scaffolds were cultured for 7 days in the experimental conditions described above. Colonized materials were evaluated throughout the culture time by confocal laser scanning microscopy (CLSM), scanning electron microscopy (SEM), and DNA content, in order to address the cell morphology, the F-actin cytoskeleton organization, and the cell proliferation.

*CLSM and SEM Observation.* For CLSM assessment, colonized scaffolds were fixed (3.7% paraformaldehyde, 15 min). Cell cytoskeleton filamentous actin (F-actin) was visualized by treating the cells with Alexa Fluor 488 Phalloidin (1 : 20 dilution in PBS, 1 h) and counterstaining with propidium iodide (1 *μ*g mL^−1^, 10 min) for cell nuclei labelling. Labelled cultures were mounted in Vectashield and examined with a Leica SP2 AOBS (Leica Microsystems) microscopy. For SEM observation, samples were fixed (1.5% glutaraldehyde in 0.14 M sodium cacodylate buffer, pH = 7.3, 10 min), dehydrated in graded alcohols, critical-point dried, sputter-coated with an Au/Pd thin film (SPI Module Sputter Coater equipment), and observed in a high resolution (Schottky) Environmental Scanning Electron Microscope (Quanta 400 FEG ESEM).

*DNA Content.* DNA was analyzed by the PicoGreen DNA quantification assay (Quant-iT PicoGreen dsDNA Assay Kit, Molecular Probes Inc., Eugene), according to manufacturer's instructions. Cultures were treated with Triton X-100 (0.1%) (Sigma), and fluorescence was measured on an Elisa reader (Synergy HT, Biotek) at wavelengths of 480 and 520 nm, excitation and emission, respectively, and corrected for fluorescence of reagent blanks. The amount of DNA was calculated by extrapolating a standard curve obtained by running the assay with the given DNA standard.

*Statistical Analysis.* Three independent experiments were performed; in each experiment, three replicas were accomplished for the DNA quantification assay and two replicas for the qualitative assays (CLSM and SEM). DNA content is presented as mean ± standard deviation (SD). Groups of data were evaluated using a two-way analysis of variance (ANOVA), and no significant differences in the pattern of the cell behavior were found. Statistical differences between experimental groups were assessed by Bonferroni's method. Values of *P* ≤ 0.05 were considered statistically significant.

## 3. Results and Discussion

### 3.1. Characterization of the Cellulose Matrix

A cellulose-based gel was lyophilized in selected conditions, and the porous matrix was created with a porosity of 80% ensuring space for vascularization and bone ingrowth.

**Figure 1:**
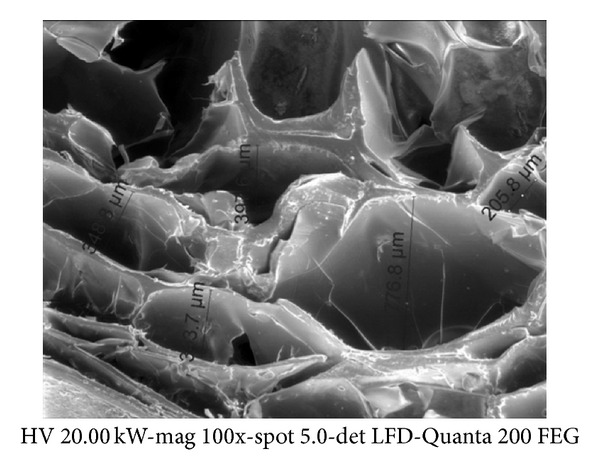
SEM image of the macroporous cellulose used in the study.

The scanning electron microscopy (SEM) micrograph revealed that the prepared matrix was composed of different pore sizes, ranging up to 780 *μ*m (Figure 1). Furthermore, the results of water retention showed that the prepared matrix could absorb more than 5 g of water per 1 g of the absolutely dried sample. This property substantiates high hydrophilicity of the matrix which reveals biocompatibility with biological systems.

### 3.2. Biomimetic Mineralization on the Cellulose Matrix

Mineral phase acts as a bonding layer to the bone; thus, it is very important to create such layer on cellulose scaffolds since cellulose itself does not have this ability.

In order to induce the mineralization process, the matrix was precalcified using three different methods: (i) treating freeze-dried cellulose samples with CaCl_2_ and (NH_4_)_2_HPO_4_ solutions; (ii) storing the carboxymethylated cellulose matrix in a saturated Ca(OH)_2_ solution; (iii) treating the lyophilized samples with a calcium silicate solution in order to introduce silanol groups and combine them with calcium ions.

**Figure 2:**
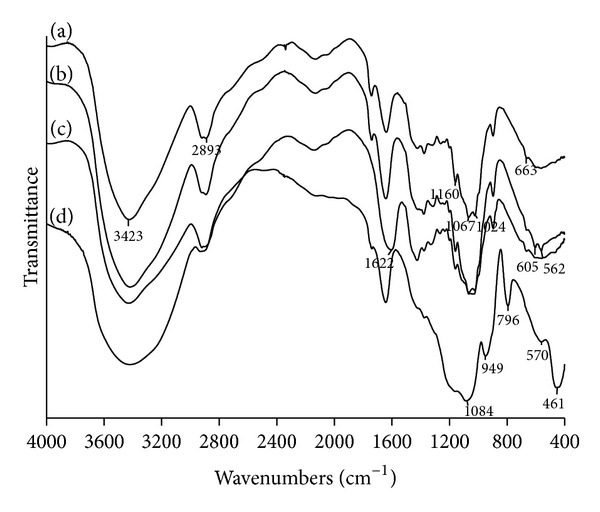
The spectra of (a) cellulose; (b) cellulose precalcified with CaCl_2_ and (NH_4_)_2_HPO_4_ solutions; (c) carboxymethylated cellulose precalcified with a saturated Ca(OH)_2_ solution; (d) cellulose pre-calcificated with the calcium silicate solution.

Figure 2 shows IR spectra of the cellulose matrix samples used for biomimetic mineralization. The broad absorption band in the 3500–3200 cm^−1^ range was assigned to the stretching vibration of the OH group of cellulose. The band, at 2893 cm^−1^, corresponds to the symmetric stretching vibration of CH_2_ groups. The absorption band due to the asymmetric C–O–C stretching vibration appears at 1160 cm^−1^. The stretching of C–O appears at 1024 cm^−1^ and 1067 cm^−1^.

After treating the cellulose matrix with CaCl_2_ and (NH_4_)_2_HPO_4_ solutions, new absorption peaks at 602 cm^−1^ and 562 cm^−1^ appeared in the spectra, confirming the formation of calcium phosphate. The spectrum of carboxymethylated cellulose (carboxymethyl groups amount 1.5 mmol/g) activated with Ca(OH)_2_ displayed an absorption band at 1622 cm^−1^, which represented the symmetric stretching vibration of a carboxyl group. Figure 2(d) shows the IR spectra of cellulose after its treatment with the calcium silicate solution. Absorption bands at 1084 cm^−1^ and 796 cm^−1^ can be seen, which correspond to the asymmetric and symmetric stretching vibrations of Si–O–Si, respectively. The band at 461 cm^−1^ is associated with Si–O–Si bending vibrations, while an absorption band at 949 cm^−1^ corresponds to the Si–OH stretching vibration. This is the result of tetraethoxysilane hydrolysis and condensation.

**Figure 3:**
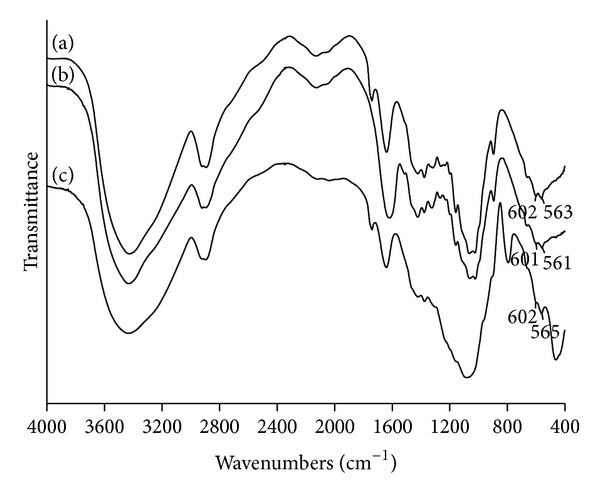
The IR spectra of (a) mineralized cellulose pretreated with calcium and phosphate ions solutions; (b) mineralized carboxymethylated cellulose pretreated with a saturated Ca(OH)_2_ solution; (c) mineralized cellulose pretreated with the calcium silicate solution.

The cellulose matrix samples were immersed in a simulated body fluid with a concentration factor of 1.5 (1.5x SBF) for up to 14 days. IR spectra of all cellulose samples after 14 days of mineralization in 1.5x SBF showed an absorption band in the 570–602 cm^−1^ range, which could be attributed to PO_4_^3−^ group (Figure 3). Apatite also gives absorption band in the 1000–1100 cm^−1^ region due to PO_4_^3−^ group. However, it should be noted that cellulose has intensive peaks in this region, which overlap with the expected peaks.

**Figure 4:**
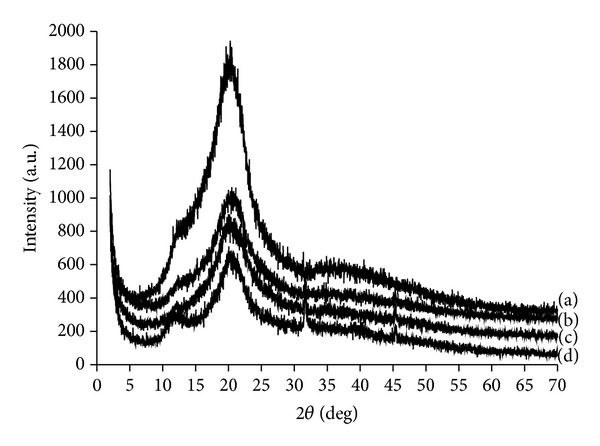
X-ray diffraction patterns of (a) regenerated cellulose; (b) mineralized cellulose pretreated with the calcium silicate solution; (c) mineralized cellulose pretreated with calcium and phosphate ions solutions; (d) mineralized carboxymethylcellulose preactivated with a saturated Ca(OH)_2_ solution.

**Figure 5:**
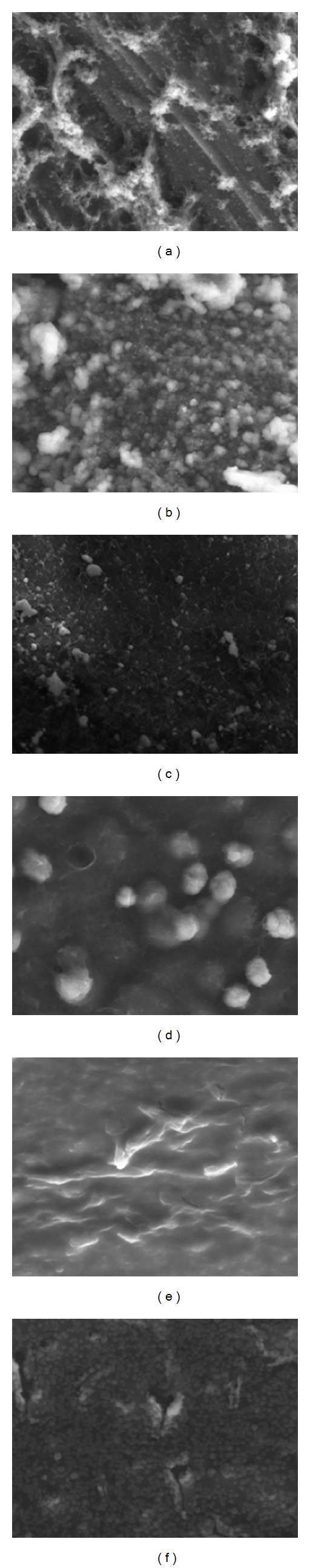
SEM micrographs of the surface of (a) the cellulose matrix treated with calcium and phosphate ions solutions then (b) soaked in 1.5x SBF; (c) the matrix activated with Ca(OH)_2_ then (d) soaked in 1.5x SBF; (e) the matrix treated with the calcium silicate solution and then (f) soaked in 1.5x SBF.

Following on, the mineralized cellulose samples were examined by an X-ray diffraction. The typical diffraction peaks of cellulose appeared at 12.4°, 20.7°, 21.1°, and 34.9° in 2*θ* (Figure 4(a)) and were also visible in the patterns of the composites. The diffraction peaks at 25.8°, 31.7°, 32.9°, 39.7°, 46.7°, and 49.6° in 2*θ* revealed the presence of calcium phosphate (Figures 4(b), 4(c), and 4(d)). Moreover, the intensity of the peaks in the X-ray diffraction pattern of carboxymethylated cellulose precalcified with calcium hydroxide (Figure 4(d)) was higher in comparison to that of other samples (Figures 4(b) and 4(c)) due to bigger crystals on the surface. This was also visible from SEM micrographs (Figure 5). Large aggregates appeared on the surface of the carboxymethylated cellulose matrix preactivated with calcium hydroxide after exposure to 1.5x SBF (Figure 5). However, small hydroxyapatite crystals covered both the cellulose pretreated with calcium and phosphate ions solutions and the cellulose matrix pretreated with the calcium silicate solution.

As well as precalcification methods, the results obtained from studies on biomimetic mineralization underlined how the type of functional groups on the cellulose surface played an important role not only for the variation in the size of the hydroxyapatite crystals but also for the deposited mass onto the surface (Table 1) like it was observed by other researchers [21, 23, 25, 26].

Our results also showed that the precalcification methods and the type of functional groups influenced the mineralization rate. As can be seen from the results, the highest mineralization rate was achieved on the carboxymethylated cellulose matrix, which was activated with the calcium hydroxide solution. Also the mass percentage of the hydroxyapatite fraction was two times larger after a few days. We assume that the higher rate of mineralization is required for the faster alteration of cellulose surface chemistry leading to an enhanced bioactivity and biocompatibility.

Matching the nanostructure of the inorganic phase of natural bone, most studies are focused on the development of scaffolds with nanohydroxyapatite. Shi et al. [27] investigated the influence of the size of hydroxyapatite on proliferation and apoptosis of osteoblast-like cells. The results showed that nanohydroxyapatite can be a better candidate in biomedical applications than microhydroxyapatite. However, we assume that nanohydroxyapatite crystals can grow and form the morphology of cauliflower in microscale (Figure 5(d)). Our findings agree with the results of Beşkardeş and Gumusderelioglu [28].

After 9 weeks of incubation, further observations showed that the matrices started to degrade (data are not presented). For these studies, it is very important that the matrix is biodegradable, as new bone would have to replace it. The degradation of the material and the whole replacing with bone tissue cell are the main objective of the current research on grafting materials. Numerous biomaterials used like scaffolds have been available for the bone regenerative techniques, unfortunately, most of these medical devices are expensive and still present unpredictable biological results [9, 16, 21, 24, 29, 30].

### 3.3. Biological Evaluation of the Mineralized Cellulose Substrates

The biological characterization of the cellulose matrix (control scaffold) and the three developed mineralized cellulose samples was conducted with MG63 human osteoblastic cells, for a period of 7 days.

**Figure 6:**
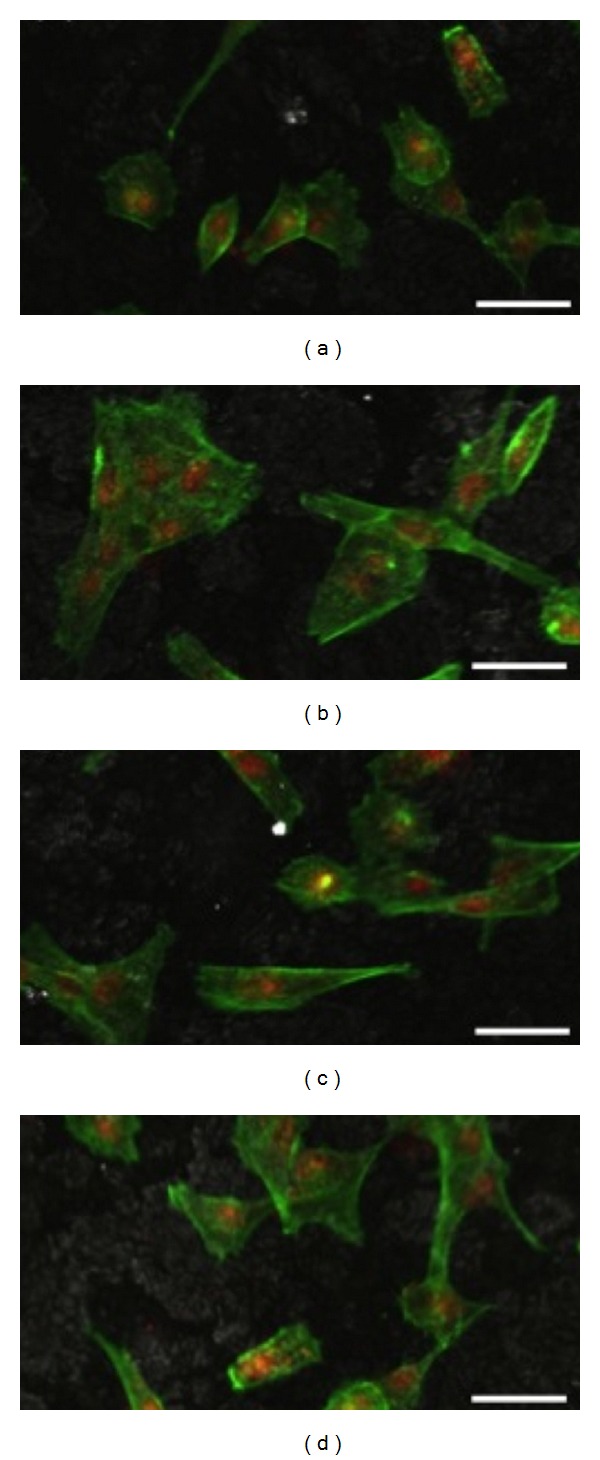
CLSM images of MG63 osteoblastic cells cultured for 24 hours over (a) cellulose matrix, (b) mineralized cellulose pre-treated with calcium and phosphate ions solutions, (c) mineralized carboxymethylated cellulose pre-treated with a saturated Ca(OH)_2_ solution, and (d) mineralized cellulose pre-treated with the calcium silicate solution. Scale bar corresponds to 40 *μ*m.

Observation of the material samples by CLSM at 1 day of culture showed well spread cells with an elongated/polygonal morphology, and establishing cell-to-cell contact, both on the cellulose matrix (control) and on the mineralized cellulose samples (Figure 6). On the assayed mineralized cellulose samples, adhered cells were found to be more expanded, with an increased cytoplasmic volume and higher number of fibrillar projections, as comparing to control. In addition, in all experimental conditions, cells exhibited a well-organized F-actin cytoskeleton, with intense staining at the cell boundaries, prominent nucleus and on-going cell division, which are signs of a healthy cellular behaviour [31]. This information is relevant in terms of the biological performance of the scaffolds, as the F-actin cytoskeleton, which is highly concentrated just beneath the plasma membrane, provides structural stability and elasticity to the cell undergoing adaptation to the substrate topography [31]. Also, the F-actin cytoskeleton is a key player in the cellular mechanotransduction mechanisms modulating complex signalling pathways essential to the subsequent stages of osteoblastic proliferation and differentiation [26, 31–33].

**Figure 7:**
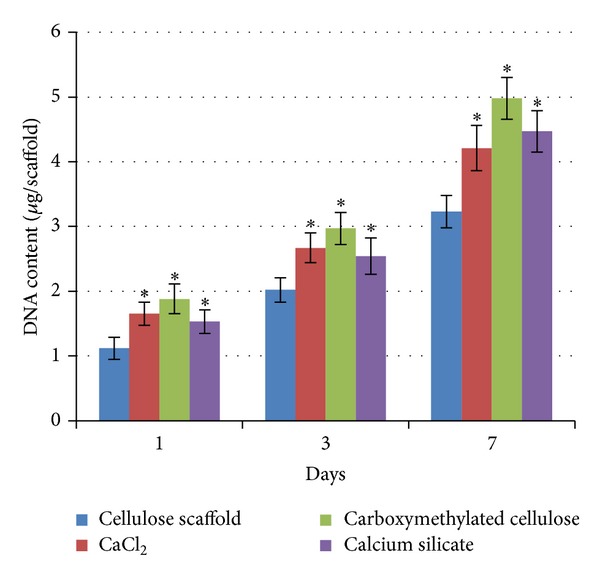
DNA content of MG63 osteoblastic cell cultures grown over the cellulose matrix and the mineralized cellulose matrices. *Significantly different from the cellulose matrix.

Assessment of DNA content showed that the cellulose matrix supports the proliferation of the osteoblastic cells, with increasing values throughout the 7-day culture period. Comparatively, it is worth to note the significantly increased values observed at day 1 for the mineralized cellulose matrices, suggesting a higher number of attached cells over these substrates. Furthermore, DNA content at days 3 and 7 was also higher in the mineralized cellulose scaffolds. Cultures grown on carboxymethylated cellulose presented, throughout the culture time, the highest values for total DNA content. However, differences among the three mineralized samples were not statistically significant. Results are summarized in Figure 7.

**Figure 8:**
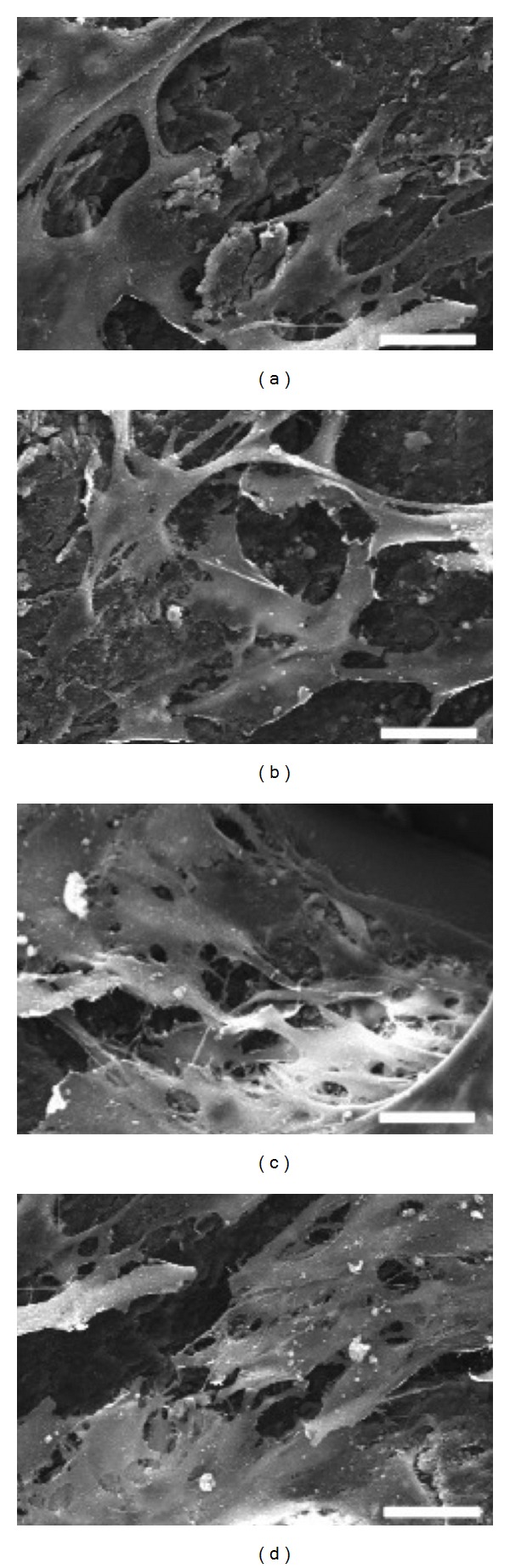
SEM images of MG63 osteoblastic cells cultured for 3 days over (a) cellulose matrix, (b) mineralized cellulose pre-treated with calcium and phosphate ions solutions, (c) mineralized carboxymethylated cellulose pre-treated with a saturated Ca(OH)_2_ solution, and (d) mineralized cellulose pre-treated with the calcium silicate solution. Scale bar corresponds to 30 *μ*m.

SEM imaging revealed that cells adhered well to the control and the mineralized cellulose matrices attaining a spreading pattern and showing a perfect adaptation to the underlying topography. Representative images are shown for cultures with 3 days (Figure 8), and the presence of an organized cell layer covering partially the material surface was evident.

The observed results ensure that the developed cellulose porous matrix is not cytotoxic and can be used in a contact with biological systems. In addition, the presence of a hydroxyapatite layer in the originally prepared cellulose matrix clearly improved cell adhesion, as shown by the higher DNA content at day 1 found in the three mineralized matrices. Also, a higher cell growth rate was evident throughout the culture time, compared to the control cellulose matrix. These results are in line with a variety of studies suggesting that cellulose is a useful scaffolding material in regenerative medicine, including in bone tissue applications, reporting the adhesion, proliferation, and differentiation of osteoblastic lineage cells [19, 34–36]. Further, there is evidence that the presence of a calcium phosphate containing layer on the surface of cellulose substrates promotes cell adhesion and growth, due to the biomimetic nature of the resulting surface [2, 19, 37, 38].

## 4. Conclusions

A highly porous cellulose matrix was successfully created by lyophilisation of a cellulose gel. The behaviour of MG-63 human osteoblastic cells on the investigated cellulose matrix confirmed it was not cytotoxic as cells were able to adhere and proliferate on it. It was found that the cellulose matrix containing silanol, carboxyl, or/and hydroxyl groups combined with calcium ions induced hydroxyapatite formation on its surface during the biomimetic mineralization. A fully covered surface was obtained during two weeks of storage in simulated body fluid at a temperature of 37°C.

Comparison of different matrix precalcification methods showed that the highest mineralization rate and larger deposited mass of hydroxyapatite were achieved on the matrix of carboxymethylated cellulose activated with a calcium hydroxide solution. The porous cellulose matrix was not cytotoxic, allowing the adhesion and proliferation of human osteoblastic cells. Comparatively, improved cell adhesion and growth rate were achieved on the mineralized cellulose matrices.

## Figures and Tables

**Table 1:** Mass increase on the cellulose matrix after the mineralization process.

Time, days	Mass increase, %		
	Matrix pretreated with the calcium silicate solution	Matrix pretreated with the calcium and phosphate ions solutions	Carboxymethylated matrix pretreated with the calcium hydroxide solution
2	2	1	4
7	3	3	7
14	6	6	12
